# Novel Technique to Overcome the Nonavailability of a Long Needle 9-0 Polypropylene Suture for Sutured Scleral Fixation of the Posterior Chamber Intraocular Lens Using a Single Fisherman's Knot

**DOI:** 10.1155/2017/2683415

**Published:** 2017-07-31

**Authors:** Yong Un Shin, Mincheol Seong, Hee Yoon Cho, Min Ho Kang

**Affiliations:** ^1^Department of Ophthalmology, Hanyang University College of Medicine, Seoul, Republic of Korea; ^2^Department of Ophthalmology, Hanyang University Guri Hospital, Guri, Republic of Korea

## Abstract

**Purpose:**

To describe a method to overcome the nonavailability of a long needle 9-0 polypropylene suture for sutured scleral fixation of the posterior chamber intraocular lens (PC-IOL) using a single fisherman's knot (SFK).

**Methods:**

First, a 10-0 polypropylene suture was passed from the sclera to the ciliary sulcus using a long needle. A 9-0 suture was tied to the unpassed portion of the 10-0 suture with an SFK. We pulled the 10-0 suture to pass the SFK through the sclera, and then we cut the knot and removed the 10-0 suture. IOL fixation with 9-0 sutures used the conventional techniques used for sutured scleral-fixated IOL. Preoperative and postoperative visual acuity, corneal astigmatism, and endothelial cell count and intraoperative/postoperative complications were evaluated.

**Results:**

An SFK joining the two sutures was passed through the sclera without breakage or slippage. A total of 35 eyes from 35 patients who underwent sutured scleral fixation of the IOL. An intraoperative complication (minor intraocular hemorrhage) was recorded in four cases. Knot exposure, IOL dislocation, subluxation, and retinal detachment were not observed.

**Conclusions:**

The SFK offers the opportunity to use 9-0 sutures for the long-term safety and may not require the surgeon to learn any new technique.

## 1. Introduction

Long-term safety of sutured scleral fixation of the posterior chamber intraocular lens (PC-IOL) implantation is an important issue for surgeons. IOL dislocations by spontaneous breakage of 10-0 polypropylene sutures have been reported [1–3]. To avoid material problems that arise from these sutures, the 9-0 polypropylene suture or Gore-Tex has been recommended as an alternative for sutured scleral fixation of PC IOL [3–5]. Curved long needles (CIF-4, Ethicon) or straight long needles (STC-6, Ethicon) are commonly used for sutured scleral fixation. However, the types of needles that can attach to a 9-0 polypropylene suture are in limited supply in Asia and many other regions; CIF-4 and STC-6 needles are not available. In addition to Ethicon products, a 9-0 polypropylene suture is available on long curved or straight needles from Visionary Medical Supplies (Madison, USA), but it is not available either in Asia or in other regions. As an alternative, a simple needle-and-hook apparatus was proposed to achieve the same effect as the STC6 needle with the 9-0 polypropylene suture [5]. Sutureless scleral fixation of the IOL can be another solution, but this technique requires vitrectomy instruments and additional surgical experience [6].

We previously suggested a simple method to restore a fractured 10-0 polypropylene suture using a single fisherman's knot (SFK) [4]. The SFK can be used to join two lines with a symmetrical structure consisting of two overhand knots, each tied around the standing part of the other; it has sufficient knot security and is simple to perform.

We introduce this clinically useful and simple SFK method to be used with 9-0 polypropylene sutures to overcome the nonavailability of a long needle 9-0 polypropylene suture.

## 2. Patients and Methods

Patients who needed the scleral fixation of IOL because of the absence of sufficient capsular structure to support the intraocular lens were included. Surgical results are retrospectively reviewed via chart review. The preoperative and postoperative visual acuity, corneal astigmatism, and corneal endothelial cell count and intraoperative and postoperative complications were evaluated. The study was approved by the Hanyang University Guri Hospital's ethics committee.

## 3. Surgical Techniques

**Figure 1:**
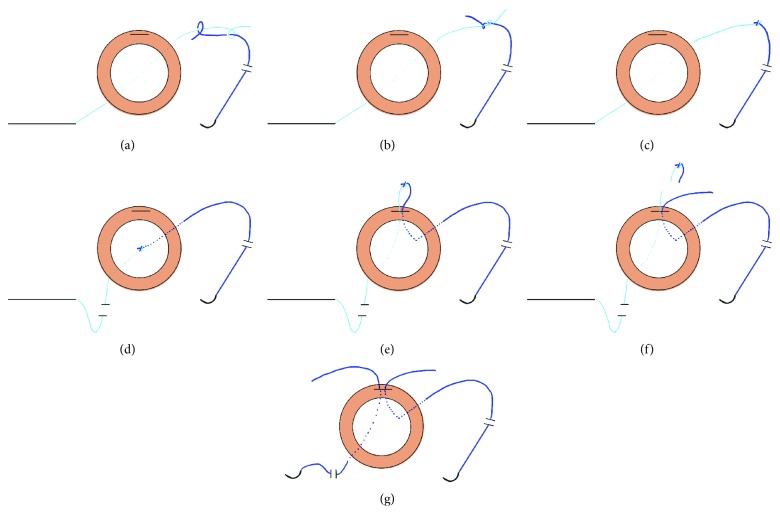
Surgical technique to prepare the 9-0 polypropylene suture using a single fisherman's knot with a 10-0 polypropylene suture: (a) overlap the ends of the 10-0 and 9-0 suture lines and make a loop with the 9-0 suture around the 10-0 suture. (b) Make a knot around the 9-0 suture and pull the standing lines in opposite directions to slide the two knots together using a single fisherman's knot (SFK). (c) After clipping the ends near the knot, (d) pull the opposite 10-0 suture to pass the SFK with the 9-0 suture. (e, f) Remove the SFK in the anterior chamber through the corneal or scleral wound for IOL insertion, and cut off the 10-0 suture with the SFK. (g) By repeating the same method, another 9-0 suture was prepared to be tied to the haptic of the IOL.

After peribulbar or retrobulbar anesthesia, localized peritomy was performed at the appropriate site. A single-arm, straight (STC-6, Ethicon), or curved needle (CIF-4, Ethicon) with a 10-0 polypropylene suture was passed through the sclera 1–1.5 mm behind the limbus using the ab externo technique, and the suture was pulled from the opposite cornea wound. The ends of the 10-0 and 9-0 polypropylene sutures were overlapped, where they were joined. The unpassed end of the 10-0 polypropylene suture was tied to the 9-0 polypropylene suture using an SFK (Figures 1(a), 1(b), and 1(c)). Next, the 10-0 polypropylene suture was pulled to allow the SFK to pass through the sclera (Figure 1(d)). The SFK was removed from the anterior chamber through the corneal or scleral wound for IOL insertion (Figure 1(e)), the 10-0 polypropylene suture was cut off at the SFK, and the 9-0 polypropylene suture was used to close the knot (Figure 1(), see Supplementary Video available online at https://doi.org/10.1155/2017/2683415, which shows the surgical procedure for Figures 1(a), 1(b), 1(c), 1(d), 1(e), and 1(f)). Repeating the same method, another 9-0 polypropylene suture was tied to the haptic of the IOL (Figure 1(g)). The surgeon can choose any IOL type that is clinically available. To avoid knot exposure, we used a Z-suture technique to fix the haptic to the sclera [7].

## 4. Results

An SFK joining 10-0 and 9-0 polypropylene sutures was passed through the sclera without breakage or slippage. A total of 35 eyes from 35 patients who underwent sutured scleral fixation of the IOL were included in this study. Seven patients were female (20%), and 28 were male (80%); the average patient age at the time of surgery was 63.2 ± 8.6 years (2 SD) (range, 51–83 years). The preoperative mean uncorrected visual acuity was 20/300. The preoperative mean corneal astigmatism was −1.30 ± 1.27 diopters, and the preoperative endothelial cell density ranged from 1266–3265 cells per square millimeter (mean, 2217.3 ± 626.2 cells/mm2). Postoperative mean uncorrected visual acuity, postoperative mean corneal astigmatism, and postoperative mean endothelial density were 20/60, −1.34 ± 0.86 diopters, and 1944.8 ± 683.2 cells/mm2, respectively (*p* < 0.001, *p* = 0.853, and *p* = 0.02).

An intraoperative complication (minor intraocular hemorrhage during needle passage) was recorded in four cases. Postoperative complications, such as knot exposure, IOL dislocation, subluxation, and retinal detachment, were not observed.

## 5. Discussion

Sutureless scleral fixation of the IOL is a new technique for overcoming the common weaknesses of sutured scleral fixation, which include spontaneous breakage, knot exposure, and IOL position problems [6, 8].

However, sutureless scleral fixation of the IOL requires posterior segment instruments and a machine for pars plana vitrectomy. Manipulation of the haptic to pull it through the sclerotomy can cause physical damage to the haptic and can compromise the long-term stability of the IOL. Intraoperative breakage or haptic deformity requires removal and reinsertion of the IOL [9]. Additionally, the risk of exposure of the haptic through the sclera cannot be ruled out. Many authors have reported that no complications related to the use of the sutureless technique arose in the short term, but long-term outcomes were not reported [10]. Even the scleral-sutured knot, which should be buried by the scleral flap, can be exposed through the scleral flap and the conjunctiva [11]. An intrastromal fixated haptic has sustained tension to the sclera, and the thickness of the sclera flap is similar to that of the flap of a sutured scleral-fixated IOL. Thus, an intrastromal haptic can erode over the sclera and conjunctiva.

Sutured scleral-fixated IOLs will continue to be a widely used option for managing IOL dislocation without IOL exchange, because dislocated IOLs are not always available in three pieces. In such cases, IOL exchanges are necessary in order to perform a sutureless scleral fixation. And it is difficult to handle the IOL haptics in the anterior chamber of severely damaged eyes. Furthermore, each surgeon has a different set of surgical skills and instruments, and we cannot ignore that operation results are affected by surgeon proficiency.

Sutured scleral-fixated IOLs with 10-0 polypropylene sutures can break spontaneously, so a thicker 9-0 polypropylene suture is more suitable for long-term stability. But the available needles are not varied enough (e.g., straight, curved, short, and long) to meet the needs of these new techniques. By using an SFK, a surgeon can use a familiar needle type, and except for making a knot, surgeons do not need additional surgical instruments or techniques. Also, ciliary sulcus fixation can be advantageous for haptic adhesion by the fibrous membrane to the ciliary body for long-term stability [12–14].

However, the larger knots made by 9-0 polypropylene sutures could erode externally through the scleral flap, exposing the patient to a higher risk of endophthalmitis. To prevent knot erosion, a “Z-suture” technique is a good solution that does not involve scleral flaps or grooves [7].

**Figure 2:**
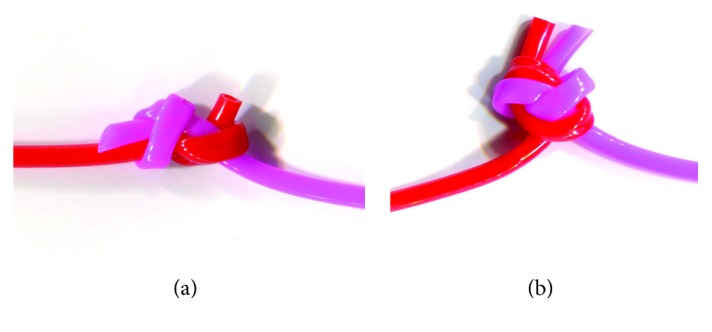
The shapes of the two different knots: the single fisherman's knot (SFK) and the double figure eight knot: (a) the SFK is a smaller knot size compared with the double figure eight knot and is aligned with the standing part. (b) The double figure eight knot is a larger knot size than the SFK and is at the right angle to the standing part.

Another simple method for making a “line-to-line knot” is to make a double figure-eight knot. However, the double figure-eight knot is bigger than the others and is at a right angle to the standing part, which increases friction as it passes through the tissue (Figure 2). However, the SFK is smaller than the double figure-eight knot and is aligned with the standing line, so it can pass through the sclera with a low friction coefficient.

The nonavailability of a long needle 9-0 polypropylene suture has restricted the application of the 9-0 polypropylene suture in Asia and other regions to prevent knot breakage in the sutured scleral fixation of IOL. The SFK is very easy to perform, is a useful method for overcoming the nonavailability of a long needle 9-0 polypropylene suture by connecting the two lines from the 10-0 and 9-0 polypropylene sutures, and is small enough to pass through the sclera without breakage or slippage.

## Supplementary Material

Supplementary video shows the steps of making an SFK and the process by which SFK passes through the sclera.
